# Case Report: Mammary and rectal metastases from an ovarian cancer: report of two cases and review of literature

**DOI:** 10.12688/f1000research.2644.2

**Published:** 2014-12-09

**Authors:** Mounia Amzerin, Camilo Garcia, Claudia Stanciu, Isabelle Veys, Ahmad Awada, Hassan Errihani, Andrea Gombos

**Affiliations:** 1 National Institute of Oncology, Université Mohammed V de Rabat, Rabat, 10100, Morocco; 2 Jules Bordet Institute, Université Libre de Bruxelles, Brussels, 1000, Belgium

## Abstract

In this paper we report two interesting cases of metastatic ovarian cancer. The first case is a patient who developed rectal and breast metastases mimicking an inflammatory breast cancer. In the second case, subclinical breast and axillary lymph node metastases were revealed by PET/CT. Metastases in the breast originating from solid tumors are extremely rare. The ovarian primitive is the fourth most common origin. The occurrence of breast metastasis is associated with an advanced disease and a poor prognosis. Their incidence is increasing since they are found more often due to better imaging techniques and to better treatment that, accordingly, improve patients’ survival. Thus, unusual sites of metastases are more and more reported. Indeed, some authors reported the occurrence of colorectal metastases from ovarian cancer. However, they remain much less frequent.

## Case 1

In November 2010, a 61 year old Moroccan housewife was diagnosed with a stage IV poorly differentiated serous ovarian adenocarcinoma (peritoneal, mediastinal and retroperitoneal lymph node metastasis). The patient had a medical history of diabetic neuropathy and hypertension. Her family history noted a sister and a niece who were respectively diagnosed with pancreatic cancer and breast cancer. A retroperitoneal lymph node biopsy was performed to obtain tumor tissue for histological diagnosis. Given the spread of the disease it was decided to administer chemotherapy first. Since she had a contraindication to taxane-based regimens because of her peripheral neuropathy, the patient received 3 cycles of Cyclophosphamide 600 mg/m ^2^ and Carboplatin AUC 5 in the first cycle and AUC6 in the subsequent cycles, every three weeks. The response assessment with PET-FDG after the third cycle showed partial response. One and a half months after the third cycle of chemotherapy, she underwent debulking surgery. The treatment was completed by two additional cycles of chemotherapy of the same combination.

Three months after the end of treatment, a CT scan showed progressive disease in the mediastinal and abdominal lymph nodes. The patient received Liposomal Doxorubicin 40 mg/m ^2^ q4w, as second line chemotherapy. The response assessment after three cycles showed disease progression. Since the patient had been asymptomatic, it was decided to wait and see.

Four months later, in January 2012, the patient presented with skin erythema and edema of the right breast. The clinical examination found ipsilateral supraclavicular lymph node swelling. The patient also complained of a diarrhea, which was resistant to standard treatments. Breast MRI showed breast and chest edema with multiple non-specific contrast uptakes giving the aspect of a homogeneous enhancement. The patient underwent sigmoidoscopy that revealed extended ulcerations of the lining of the rectum ( Figure 1A). The breast and rectal biopsies showed a positive staining of PAX8 by immunohistochemistry ( Figure 1B). Both lead to a diagnosis of metastasis from the known serous ovarian neoplasia. The patient was treated by topotecan as third line chemotherapy Topotecan 4 mg/m ^2^ days 1, 8, 15; every 28 days. Unfortunately the disease progressed dramatically after two cycles. The patient died in March 2012, 18 months after the initial diagnosis, 3 months after the diagnosis of the breast metastasis.

**Figure 1A.**
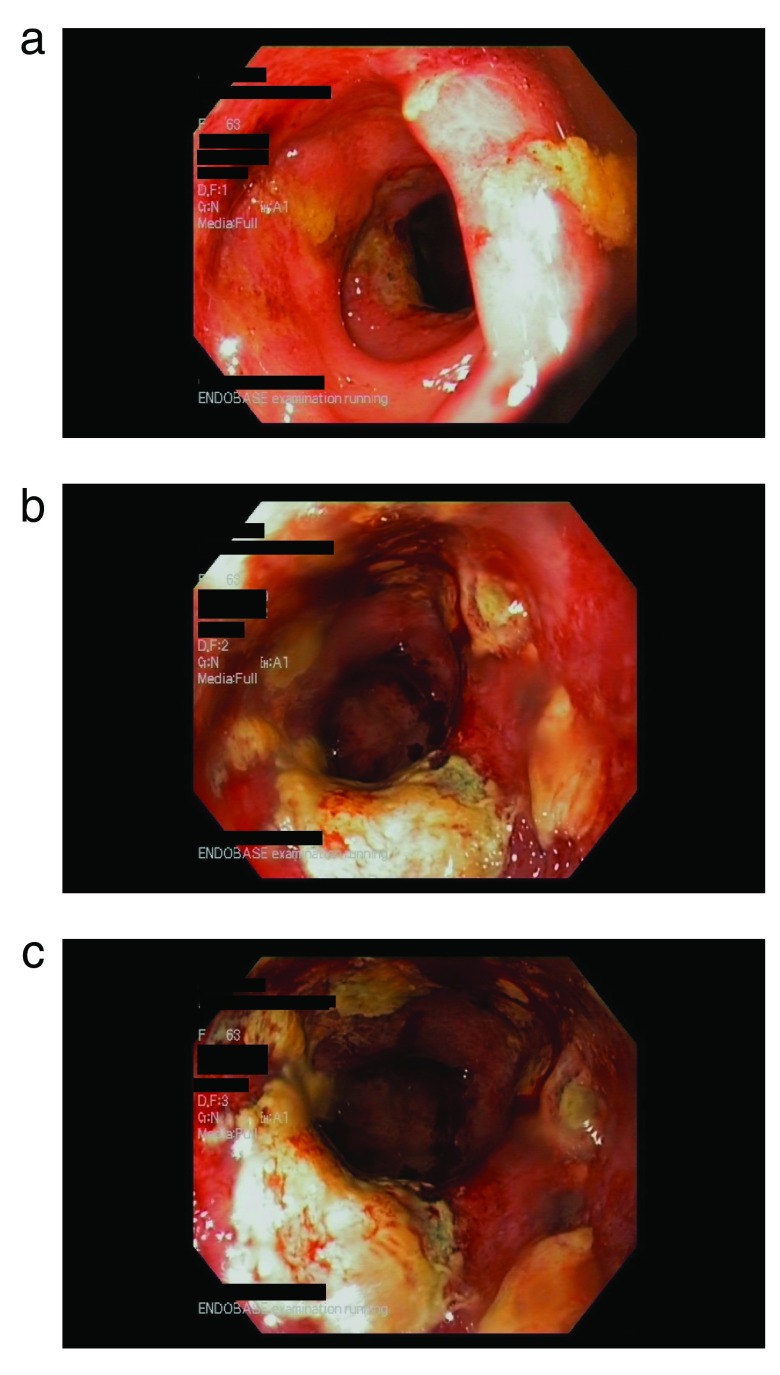
Rectal endoscopy. Ulceration in the lining of the rectum corresponding to metastases from the ovarian primary.

**Figure 1B.**
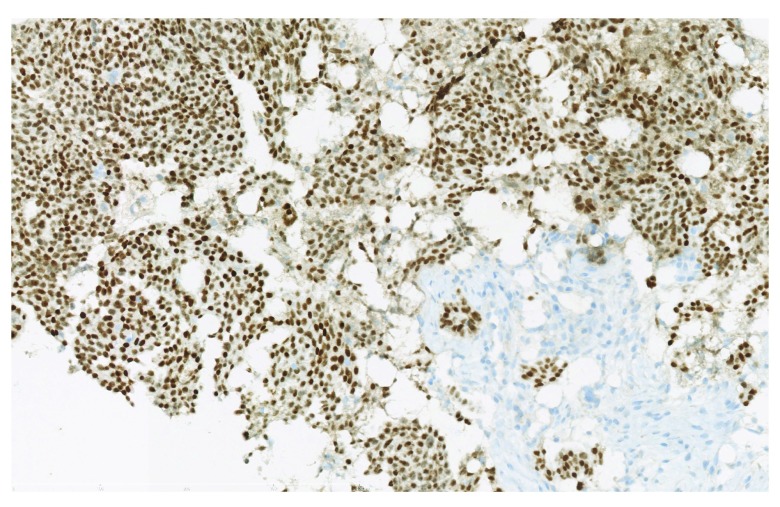
Biopsy of the breast metastasis. 20× magnification. Positive staining PAX8 on immunohistochemistry: Nuclear staining dark brown in tumor cells of ovarian origin within the mammary stroma stained blue.

## Case 2

A 52 year old Caucasian patient was diagnosed with a cyst of the right ovary. She had a medical history of a herniated disc and cholecystectomy. She had no family history of cancer. During an exploratory laparoscopy, a peritoneal carcinomatosis was found. The patient underwent a suboptimal debulking surgery consisting of hysterectomy and bilateral salpingo-oophorectomy. The histopathological examination showed a serous papillary adenocarcinoma of the ovary, classified as at least FIGO stage IIIc. She was then treated with 3 cycles of combination chemotherapy consisting of Paclitaxel 175 mg/m ^2^ and Carboplatin AUC 6 q3w. The patient underwent a second surgery one month after the third cycle of chemotherapy, to perform omentectomy and lomboaortic node dissection. From 25 resected lymph nodes, 14 were positive for tumor cells. The patient received three additional cycles of the same combination of chemotherapy.

Seventeen months later, the patient consulted her doctor for nausea and abdominal pain. The physical examination was strictly normal. The blood test showed an increased Ca125 tumor marker. The PET/FDG revealed recurrence of the cancer in the peritoneum. Moreover, it showed a Fludeoxyglucose (FDG) uptake at PET-CT in the right breast and the ipsilateral axillary lymph node suggesting a primary breast cancer ( Figure 2A). Both ultrasound and MRI confirmed multiple lesions in the right breast. An ultrasound guided biopsy showed metastasis from ovarian cancer corresponding to a PAX8 overexpression on the histological examination ( Figure 2B). The patient received a second line chemotherapy consisting of Gemcitabine 1000 mg/m ^2^ d1, d8 and Carboplatin AUC 4 d1 q3w. After six cycles, a CT scan showed partial response according to RECIST criteria ^1^ whereas metabolic assessment by PET/FDG-CT revealed a stable disease. The reassessment three months after the end of chemotherapy showed progressive disease. The patient received Liposomal Doxorubicin 40 mg/m ^2^ q4w. After six cycles, the disease progressed. The patient received third line chemotherapy consisting of weekly Paclitaxel 80 mg/m ^2^ d1, d8, d15 q4w. The last assessment of tumor response to Paclitaxel in May 2014 showed stable disease.

**Figure 2A.**
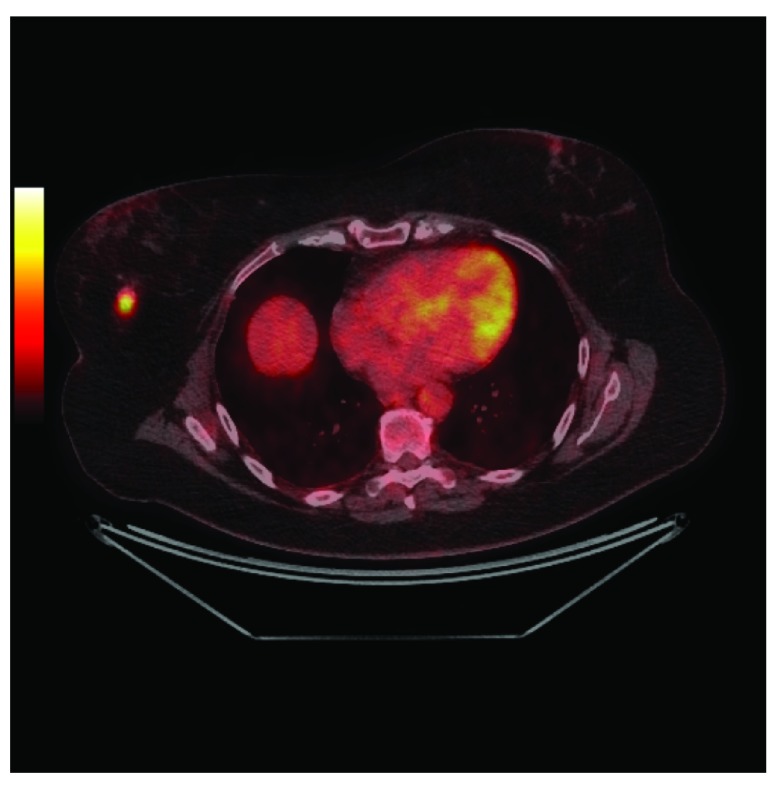
FDG PET/CT image of the breast metastasis. Fused FDG PET/CT images showing an intense focal FDG uptake in the right breast coinciding with a dense breast nodule on CT images. The hot iron FDG PET scale represents intensity of FDG uptake, varying from black at the weakest intensity to white at the strongest.

**Figure 2B.**
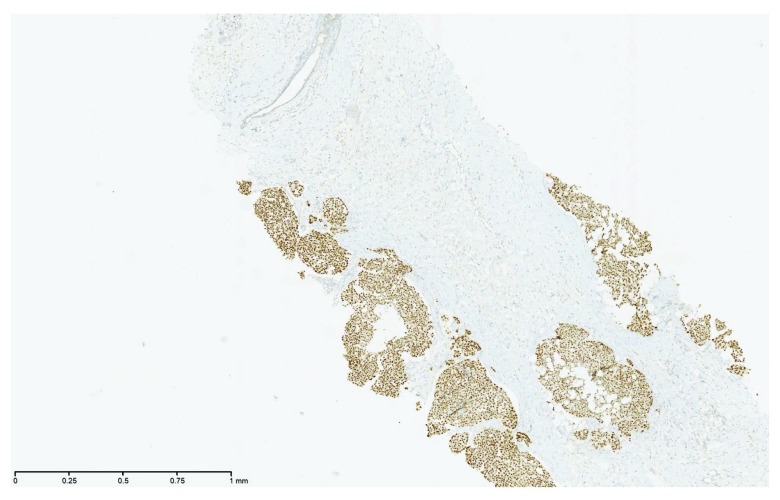
Biopsy of the breast metastasis. 4× magnification. Positive staining PAX8 on immunohistochemistry: Positive staining PAX8 on immunohistochemistry: Nuclear staining dark brown in tumor cells of ovarian origin within the mammary stroma stained blue.

## Discussion

Breast metastases from a non-mammary origin remain anecdotal. Some retrospective studies of patients with metastatic cancers from different primaries, report an incidence ranging from 0.1 to 1.3% ^2– 4^. Over a period of 20 years, *Delair et al.* recorded only 85 cases ^5^.

Most often, metastases in the breast originate from lymphomas and melanomas. Among the gynecological primitives, ovarian serous carcinoma is the most frequent ^5, 8^, which is the case in our two patients. Samples from 825 breast cancers were studied by high throughput sequencing techniques and compared with basal like breast cancer, using data originating from the Cancer genome Atlas ^9^. Interestingly, it was discovered that basal-like tumors share the same driving mutations, i.e. they are genetically similar to high grade serous ovarian carcinomas ^9^. We are not sure if this could explain the fact that serous ovarian carcinoma is the one that, in comparison with other gynecological primaries, is the most frequently responsible for metastasis in the breast. Unfortunately, in the case of our patients, we were not able to perform deep gene sequencing analysis since we haven’t archived frozen tissue.

Fewer than 50 similar cases have been reported since 1907, when A. Sitzenfrey first described a patient diagnosed with breast metastases of ovarian origin ^10, 11^. The incidence of extra mammary metastases is increasing due to the improvements in treatment leading to longer survival. Thus, unusual sites of metastases are more and more reported. In addition, the frequent use of modern imaging techniques like PET-CT leads to the detection of subclinical lesions. When the PET-CT was used for the work-up among patients with FIGO stage IIC-IV ovarian cancer, a supradiaphragmatic lymph node disease was detected in 67% of cases even if conventional imaging had showed no metastasis ^12^. This was also the case in both patients described here.

The most frequent metastatic sites from ovarian cancer are the peritoneum, omentum, colon, lungs and lymph nodes. The breast metastases are very uncommon. They can occur by both hematogenous and lymphatic spread ^13^.

When breast metastases from an extra-mammary origin occur, which is exceptional, they are generally diagnosed in patients with a known metastatic disease. Thus, the diagnosis of mammary metastasis from an ovarian origin is often evoked, especially when their occurrence is metachronous. When the diagnosis of a breast tumor and an ovarian primary are made simultaneously, a breast biopsy should be performed in order to exclude a primary breast cancer diagnosis. That is because the association of two distinct primaries is not rare especially for patients carrying BRCA1/2 mutations. However, all clinical presentations are possible. A case of breast metastases announcing an ovarian cancer has also been previously reported ^14^.

Unlike primary breast cancer, extra mammary metastases are usually superficial, mobile, well circumscribed and painless ^10, 15^. Although, they can have a clinical presentation mimicking an inflammatory breast cancer, these cases are even rarer. To our knowledge, only seven cases of metastatic ovarian cancer to the breast have been described so far ^13^.

The mean interval between the diagnosis of ovarian cancer and the breast metastasis (if it occurs) is between 2 and 3 years ^8, 16^. Although shorter term has also been reported ^13^. Our two patients developed secondary breast disease during the second year after diagnosis.

Imaging techniques could help diagnosing breast metastases. On a mammography, they are usually well circumscribed and dense without spiculations or microcalcifications ^13^. However, serous histology can be associated with microcalcifications so differential diagnosis can sometimes be difficult ^8, 17^. However, these imaging techniques are unable to formally distinguish a primary breast cancer from a metastasis.

A biopsy remains the only way to confirm the diagnosis. The classical histopathological examination with Hematoxylin and eosin stain could be inconclusive, since both ovarian and breast cancers have papillary, glandular or solid architecture. Standard immunohistochemical analysis is often unhelpful, because both ovarian and breast primary tumors are usually CK7 positive and CK20 negative. In addition, both cancers could express or not, estrogen and/or progesterone receptors. So far, PAX8 is the only known marker that can reliably make the differential diagnosis between breast cancer and ovarian cancer, since gynecological cancers are PAX8-positive whereas breast cancers are PAX8-negative ^18^. Wilm’s Tumor 1 Receptor WT1 has also been described as an interesting marker to make the differential diagnosis between a metastasis from ovarian origin and a breast primary, although some breast cancers have been found positive for WT1 ^18, 19^. For our patients, the pathologist used PAX8-staining to confirm the diagnosis of metastasis from ovarian origin.

Differential diagnosis is crucial in order to define the appropriate treatment for the patient. If the diagnosis is primary breast cancer, then the treatment would be a combination of surgery, chemotherapy, biological agents (such as anti-Her2 therapy her2 positive tumors, and antiangiogenic therapy), hormonal therapy if the cancer expresses hormonal (estrogen, progesterone) receptors and/or radiotherapy if indicated. In the case of breast metastases, surgery should not to be the first option, since in metastatic ovarian cancer the chemotherapy is the main course of treatment.

Unfortunately, breast metastasis of an ovarian cancer indicates an extensive disease. The prognosis is poor. Previous studies report survivals ranging between 13 days and 3.5 years ^10^. However, rare cases of longer overall survival have also been observed ^10, 14^. Our first patient died only 2 months after the diagnosis of the breast metastasis.

Finally, the case of our first patient is also interesting regarding the presence of histologically confirmed rectal metastases. Only 5 cases of colorectal metastases from an ovarian cancer have been reported, mainly in the clear cell carcinoma subtype ^20– 24^. Thus, in this paper, we have described the sixth case and the first case with both breast-and rectal metastases from a serous papillary ovarian carcinoma.

## Conclusion

To our knowledge, this is the first case reported in literature that describes both rectal- and breast metastases mimicking an inflammatory breast cancer, that derived from a serous papillary ovarian cancer. Our second case illustrates the role of PET-CT in detecting subclinical metastases, which leads to an increase in the diagnosis of uncommon sites of secondary dissemination of ovarian cancer. The differential diagnosis between a primary and a secondary breast cancer is crucial to provide the appropriate treatment. Unfortunately, the occurrence of breast metastases in an ovarian carcinoma is linked to an extensive disease and a poor prognosis.

## Patient consent

The consents of the first patient’s daughter and of the second patient were obtained before submitting to publication.
