# Supplementary material for: Case Report: Mammary and rectal metastases from an ovarian cancer: report of two cases and review of literature
Source: F1000Res. 2014 Dec 9;3:255. Originally published 2014 Oct 28. [Version 2] doi: 10.12688/f1000research.2644.2 (PMC4275024; doi:10.12688/f1000research.2644.2)
Supplement: Peer review history

# Peer review: Referee response for version 1

**DOI:** 10.5256/f1000research.2864.r6791

**Published:** 2014-12-01

**Authors:** Véronique D'Hondt

**Affiliations:** 1 Institut de Recherche en Cancérologie de Montpellier, Montpellier, France

Two interesting cases of unusual evolution of ovarian carcinoma.

Well written paper with a good discussion and a large review of literature.

Congratulations to the authors!

Minor comments:

page 2; case 2; 12th line: **lomboaortic **instead of lumboaortic

page 3; discussion; end of 2nd paragraph: **we haven't **archived

page 3; discussion; 3rd paragraph: modern **imaging **techniques

page 4; 4th paragraph: help **diagnosing**

I have read this submission. I believe that I have an appropriate level of expertise to confirm that it is of an acceptable scientific standard.

# Peer review: Referee response for version 1

**DOI:** 10.5256/f1000research.2864.r6546

**Published:** 2014-10-31

**Authors:** Daniel Vorobiof

**Affiliations:** 1 Department of Medical Oncology, Sandton Oncology Centre, Johannesburg, Gauteng, South Africa

Interesting case reports. I agree with the authors that as imaging is becoming more accurate and available, unusual sites of metastatic disease are diagnosed, contributing to our understanding of the evolution of different malignant diseases.

I have read this submission. I believe that I have an appropriate level of expertise to confirm that it is of an acceptable scientific standard.
